# Hearing Outcomes in Children with Unilateral Hearing Loss. The Benefits of Rehabilitative Strategies: Preliminary Results

**DOI:** 10.3390/audiolres15020037

**Published:** 2025-04-02

**Authors:** Rita Malesci, Carla Laria, Giovanni Freda, Valeria Del Vecchio, Antonietta Mallardo, Nicola Serra, Gennaro Auletta, Anna Rita Fetoni

**Affiliations:** 1 Department of Neuroscience, Reproductive Sciences and Dentistry, Audiology Section University of Naples Federico II, via Pansini 5, 80131 Naples, Italy; dott.giovannifreda@gmail.com (G.F.); toniam951@gmail.com (A.M.); nicola.serra@unina.it (N.S.); auletta@unina.it (G.A.); annarita.fetoni@unina.t (A.R.F.); 2 Hearing and Balance Unit, Department of Head and Neck, Federico II University Hospital, via Pansini 5, 80131 Naples, Italy; valeria.delvecchio@unina.it

**Keywords:** unilateral hearing loss, single side deafness, amplification, children, hearing aid, cochlear implant

## Abstract

**Background/Objectives**: Unilateral hearing loss (UHL) is a relatively common disability condition comprising around 20–50% of all congenital hearing loss (HL). The adverse effects of UHL affect the typical development of auditory function with implications for communication, speech and language acquisition, academic development and quality of life. Current literature suggests an early intervention treatment in order to avoid developmental delays, but there is a lack of evidence about the effectiveness and use of hearing devices. The purpose of the present study was to evaluate the benefits of rehabilitative strategies such as hearing aid (HA) and cochlear implant (CI) in UHL children by exploring audiological and parent-reported outcomes. **Methods**: A total of 18 UHL children, between the ages of 3 and 17, were enrolled in the study designed as a prospective longitudinal study from July 2023 to July 2024. All children were evaluated for speech perception in quiet and noise and subjective benefits before and after rehabilitative treatment with HA in 15 (83.3%) children and with CI in 3 (16.7%) children. **Results**: The evaluation of audiological outcomes in children with UHL, based on assessment of aided sound field thresholds and speech perception scores assessment versus unaided, shows improvements in audiometric thresholds and how the hearing devices adequately support listening and spoken language. Scores with hearing devices were significantly higher than baseline-only scores when averaging both SSQ and CHILD questionnaires, pointing to an overall rehabilitative benefit. **Conclusions**: Rehabilitative interventions, particularly HA and CI, offer notable benefits when introduced early, but achieving optimal outcomes requires a multidisciplinary and individualized approach.

## 1. Introduction

Unilateral hearing loss (UHL) is a relatively common condition, accounting for approximately 20–50% of all cases of congenital hearing loss (HL) [1,2,3,4,5]. The prevalence of early-identified UHL is estimated to range from 0.6 to 1 per 1000 newborns [3,4,6,7]. More than half of UHL cases may emerge later in childhood [4,7] and, over time, 20% to 40% of congenital UHL cases progress to bilateral HL [4,5,7]. Currently, the World Health Organization (WHO) defines unilateral hearing loss (UHL) in adults as a pure tone average (PTA) of hearing thresholds at 0.5, 1.0, 2.0, and 4.0 kHz, with a threshold of 35 dB or greater in the worse ear and <20 dB in the better ear [8]. However, the WHO does not provide a standardized definition for UHL in children, leading to variability in criteria across studies [5]. Therefore, it is possible to declare any degree of permanent HL in one ear (pure-tone average [0.5, 1.0, 2.0 kHz] > 15 dB for children), regardless of etiology, with normal hearing in the opposite ear [9].

HL can range from mild to profound and has different audiometric configurations [10,11]. Specifically, single-sided deafness (SSD) refers to profound sensorineural HL or nonfunctional hearing in one ear, while the other ear maintains normal hearing [11,12].

SSD may result from different etio-pathological mechanisms and may vary in presentation between pediatric and adult populations [13].

Etiology is more often related to congenital cytomegalovirus, followed by structural anomalies, such as enlarged vestibular aqueduct and cochlear nerve deficiency. Only a small number of cases are due to genetic causes.

The variability in UHL etiology underscores the complexity of its diagnosis. Early identification proves to be particularly challenging in cases where the loss goes unnoticed until a child begins exhibiting developmental delays. This diagnostic delay can significantly hinder intervention outcomes, as auditory deprivation disrupts the precise neural mechanisms that govern auditory and language processing during critical development periods [14].

Historically, the diagnosis was often at school age before the implementation of universal newborn hearing screening, which improved early detection of hearing impairment. However, nowadays, most children with mild HL and those with late-onset or acquired HL are diagnosed later because of limits limitations of universal newborn hearing screening [11,15], highlighting the need for broader awareness among clinicians, educators and families.

Moreover, the historical belief was that children with UHL experience negligible disadvantages because they have access to speech and develop spoken language without intervention [16]. Nevertheless, children with UHL cannot take advantage from binaural benefits, such as sound localization and when listening to noise, and encounter difficulties in accessing relevant acoustic information from natural listening. These adverse effects of UHL affect typical development of auditory pathways and auditory function with implications for communication, speech and language acquisition, and academic development for at least some children [16,17,18], such as grade repetition and requirement of additional educational services, and deficits in behavior and quality of life [16,19,20,21,22].

Therefore, nowadays, current literature suggests early intervention treatment in order to avoid developmental delays and learning disorders [18,23], although there is a lack of evidence about the effectiveness and use of hearing devices [24,25,26,27,28]. Rehabilitative options, such as HA and CI, offer potential pathways for reducing the adverse effects of UHL, though their success is contingent upon timely and consistent use. The clinical in-decision to treatment is due to the inconsistent HA use reported, suggesting nonuse or in-consistent use in 30–50% of these children [25,28,29]. However, most of these data are from children who did not undergo newborn hearing screening and were identified later in childhood.

Results of recent literature show that children may experience improved speech perception in quiet and noise, as well as sound source localization. The possible role of CI in improving the quality of life of children with unilateral deafness is still controversial [30,31], in particular, children with acquired SSD and shorter durations of deafness report experiencing greater advantages and were less likely to become nonusers of implant devices.

However, despite advancements in technology and a better professional understanding of UHL in children, families often fail to recognize the potential impacts of UHL on their children and do not always accept the available rehabilitation options.

Therefore, a comprehensive assessment based on measures that focus on speech-in-noise ability and on sound localisation are recommended to assess the need for hearing assistive technologies that enhance the signal-to-noise ratio and to determine degree of difficulty [9].

Currently, a clinically feasible approach to measure sound localisation capabilities is based on adoption of surveys or questionnaires, such as *Children’s Home Inventory for Listening Difficulties* (CHILD) by Anderson and Smaldino 2007 [32] and the *Speech and Qualities of Hearing Questionnaire* (SSQ parent) by Galvin and Noble 2013 [33]. In addition, these functional assessment tools explore the possible difficulties of UHL children in daily life and help the parents to have more consciousness of the disability and to better observe their child. The information revealed by the questionnaires can assist a parent in understanding the consequences of newly identified HL, which has subtle, and often mislabeled, communication effects. Given these challenges, evaluating the benefits of hearing rehabilitation strategies in children with UHL is crucial for guiding clinical decision-making and improving outcomes. Therefore, counseling about tools with parents to promote hearing rehabilitation and verifying the benefits of hearing devices during the follow-up are also useful. Thus, the opportunities for early hearing rehabilitation is currently challenging, regarding impact on child and family.

The purpose of the present study was to evaluate the benefits of rehabilitative strategies, such as HA and CI, in UHL children by exploring audiological and parent-reported outcomes through aided sound field thresholds, aided speech perception assessment in noise and parental questionnaires.

## 2. Materials and Methods

The study was designed as a prospective longitudinal study.

All 18 participants included in the study had UHL and were recruited from the Audiology Unit at the Department of Neuroscience, Reproductive Sciences, and Dentistry, University of Naples Federico II. The recruitment took place at the Regional Reference Center for the Early Diagnosis of Deafness in the Campania Region, during the period from July 2023 to July 2024.

The study was carried out in accordance with the Declaration of Helsinki and was approved by the Ethical Committee recommendation under protocol code 271/22 on 6 June 2023. All data were treated in accordance with current legislation on privacy (EU 679/2016).

Children aged between 3 and 17 years with a diagnosis of unilateral sensorineural HL were eligible and audiological rehabilitative treatment was suggested to all their families.

The diagnosis of UHL was confirmed in all enrolled patients by a unilateral hearing threshold of ≥25 dB nHL and ≤20 dB in the contralateral, normal hearing ear.

The inclusionary criteria were diagnosis of UHL, acceptance for rehabilitative treatment with HA or CI and age ≤ 18 years. Exclusionary criteria were bilateral HL, congenital malformations of the outer ear, otitis media with effusion and developmental delay precluding potential to participate in study outcome measures, and age > 18 years.

During audiological entry-level evaluation, an expert pediatric audiologist made an accurate investigation of clinical history including HL etiology, otologic examination findings and previous diagnostic tests. The diagnosis of UHL was confirmed through instrumental evaluation using pure-tone audiometry.

Moreover, we investigated the onset of UHL by considering the age at diagnosis and the results of previous instrumental evaluations. HL was classified as prelingual if the child was either born deaf or lost hearing early in childhood, before acquiring language by the age of 3. If not, HL was considered as late onset and post-lingual.

Rehabilitative treatment was then recommended, either with HA or CI. Each child was fitted with a behind-the-ear HA with a standard ear-hook and a custom earmold on the poorer ear fitted with DSL v. 5.0. Cochlear implantation surgery was considered only for selected patients with profound hearing loss, who had not benefited from previous HA use.

Therefore, the criteria adopted for implantation were unilateral profound hearing loss rehabilitated with HA for at least 6 months without benefits.

Additionally, each subject’s primary caregiver completed two questionnaires, the CHILD and SSQ, to evaluate the extent of hearing disability. All families completed the questionnaires at the initial appointment and again at 6 months following the commencement of the rehabilitation program. At the follow-up appointment, caregivers completed the questionnaires twice, providing feedback on the outcomes both with and without the use of hearing devices. At the same time, all children underwent audiological assessment, which included pure tone audiometry, speech audiometry, aided sound field, and aided and unaided simplified Italian matrix (SiIMax version 2.0) test with and without device (Madsen^®^ Astera2, Natus, Middleton, WI, USA), and we verified the daily use of the device, collecting its data logging.

### 2.1. Audiological Assessment

The same expert audiologist technician carried out all procedures in a soundproof and faradized room.

Pure tone audiometry (Madsen^®^ Astera2, Natus) was performed for both air and bone conduction and the PTA was measured for the frequencies 0.5, 1.0, 2.0 and 4.0 kHz. Aided sound field audiometry was performed with the hearing device positioned on the affected ear. On the contralateral normal hearing ear, masking narrow band noise was administered with headphones at 50 dB nHL over the hearing threshold.

Degrees of HL were classified according to PTA of unaided pure tone audiometry. Normal hearing is indicated as 20 dB nHL or better, mild as 25 to 40 dB nHL, moderate as 41 to 60 dB nHL, severe HL as 61 to 80 dB nHL and profound HL greater than 80 dB nHL [34].

Speech audiometry (Madsen^®^ Astera2, Natus) was performed in quiet for air conduction administration, masking white noise in the contralateral normal ear. Aided speech audiometry was performed in free field conditions with the hearing device on the affected side and masking white noise by means of headphones on the contralateral normal hearing ear at 50 dB nHL over the hearing threshold. We collected speech recognition threshold (SRT), the minimum hearing level for speech at which an individual can recognize 50% of the speech material.

The SiIMax (Madsen^®^ Astera2, Natus) is a speech audiometry system in background noise with automatically adapted signal to noise ratio using 14 randomized lists of three-word sentences with the same structure (number, adjective, noun), semantically unpredictable and administered after a training session to minimize the learning curve. The background noise is presented at a fixed 65 dB sound pressure level, while the speech material is actively adapted by the software. The result of the test is SRT at SiIMax, a score representing the signal-to-noise ratio in dB at which the patient can recognize 50% of the speech material.

### 2.2. Questionnaire

The SSQ, based on the SSQ developed for adults [35], was modified and adapted for parents of children with impaired hearing [33]. The SSQ permits to information on three areas of a child’s daily functioning when listening with and without hearing device: speech understanding in quiet, in background noise, in groups, in reverberant environments, and on the telephone; spatial hearing as the perception of the position, movement and distance of sound sources; quality of hearing as the identification of sounds and voices, ease of listening, and segregating sounds. The purpose of this questionnaire is to gather information on three key aspects of a child’s daily functioning when using cochlear implants and/or hearing aids: section A (Speech), which assesses speech comprehension in quiet environments, background noise, group settings, reverberant spaces, and during telephone conversations; section B (Spatial Hearing), which evaluates the ability to perceive the location, movement, and distance of sound sources; and section C (Qualities of Hearing), which focuses on sound and voice identification, listening ease, and the ability to segregate sounds. The questionnaire was translated and administered in Italian to parents. They completed the scale face-to-face with the clinician in order to ensure that the questions were correctly interpreted and indicated a score of the child’s listening skill on a scale from 1 to 10 for each situation described. A higher score designates a better hearing quality.

The CHILD questionnaire [32] is a 15-part questionnaire completed by the care-giver and requires them to score (from 1 to 8) how likely their child will be able to hear in different scenarios. The result is reported as an average score of 15 responses.

### 2.3. Statistical Analysis

Data were presented as number and percentage for categorical variables, and continuous data were expressed as the mean ± standard deviation (SD) or median and interquartile range (IQR). The test for normal distribution was performed using the Shapiro–Wilk test. The *t*-test was used to test the differences between two means of paired data. Alternatively, the Wilcoxon test was used if the distributions were not normal. Additionally, since the sample sizes for each subgroup were smaller, we performed the power analysis for each statistical test using the effect size. Particularly, the effect size was computed by phi coefficient for categorical variables, by *η*^2^ and *r* for non-parametric test (Mann–Whitney test and Wilcoxon signed-rank test, respectively), and by Cohen’s *d* index (paired *t*-test).

Finally, all tests with *p*-value (*p*) < 0.05 were considered significant. Statistical analysis was performed using the Matrix Laboratory (MATLAB) analytical toolbox version 2008 (MathWorks, Natick, MA, USA) for Windows 10 at 32 bits.

## 3. Results

A total of 18 children with UHL, including 11 (61.1%) males and 7 (38.9%) females, who attended our tertiary-level referral audiology center, met the inclusion criteria. All participants were enrolled in the study after their families accepted the rehabilitative treatment. The mean age of the study population at recruitment was 8.6 ± 4.1 years while the age at diagnosis of UHL was 4.1 ± 5.0 years (range between 1 month and 17 years) and the mean time elapsed between HL diagnosis and treatment was 4.1 years. Table 1 provides a summary of demographic and audiological characteristics observed in our sample.

The diagnosis of UHL was early (prelingual) in 8 patients (3 Cytomegalovirus, 1 genetic, 4 unknown) and later (post-lingual) in 10 children (2 genetic, 2 malformations, 1 cholesteatoma, 5 unknown). Among prelingual children, 4 had unilaterally failed the universal neonatal hearing screening while 4 received a later diagnosis during audiological surveillance (1 baby infected by Cytomegalovirus) or later because of language delay.

All patients completed the protocol. The side of UHL was right in 7 patients (38.9%) and left in 11 patients (61.1%). The mean hearing threshold of UHL was 77.7 ± 23.3 dB nHL and the degree of HL was mild in 1 (5.6%) child, moderate in 3 (16.7%), severe in 6 (33.3%), profound in 8 (44.4%). Moreover, 9 children received the diagnosis of single-sided deafness with a hearing threshold higher than 70 dB nHL. In the contralateral ear, the mean hearing threshold was 14.5 ± 4.8 dB nHL. In our sample 15 children accepted the rehabilitation with HA and 3 patients with profound HL, and no benefits from HA were selected for the treatment with CI. The latter were a child 12 years old (144 months) with congenital idiopathic UHL, and two late onset hearing loss respectively 8 (96 months) and 16 years old (192 months). The rehabilitation with HA started later in congenital year loss at the age of the recruitment to the study, while both late onset hearing losses used HA since they had received a diagnosis of UHL.

The mean daily usage of hearing devices (HA or CI) was 8.3 h. In Table 2, we have reported the results of the age at diagnosis and at the rehabilitation and audiological assessment with pure tone audiometry as PTA, speech audiometry as SRT and SiIMAX as SRT with and without the HA/CI.

The mean PTA of aided sound field pure tone audiometry was 48.4 ± 14.5 dB nHL, and significantly improved (*p* < 0.0001) compared to unaided threshold at the same time (79.2 ± 23.1 dB nHL) (Table 3).

All tests were performed on 18 children, except where the number is specified.

The speech audiometry was administered in 12 subjects out of 18 patients and SiIMax test in 13 patients out of 18, because of the difficulties of performing the test in very young children. At the speech audiometry, the mean SRT with hearing device was 41.3 ± 17.7 dB and 79.9 ± 29.3 dB without device. The mean score of SRT at SiIMax test with the device was −3.2 ± 2.2 and without the device was −2.6 ± 2.5.

From power analysis, we observed that for SRT at SiIMax test the significant result had low probability of bias due to small sample size.

Concerning the questionnaires, all families answered both SSQ and CHILD and confirmed a global improvement in performance after the rehabilitative treatment.

In detail, we compared all the results of the children after the rehabilitative treatment with and without hearing device, performing both classical statistical analysis and power analysis using effect size.

**Figure 1:**
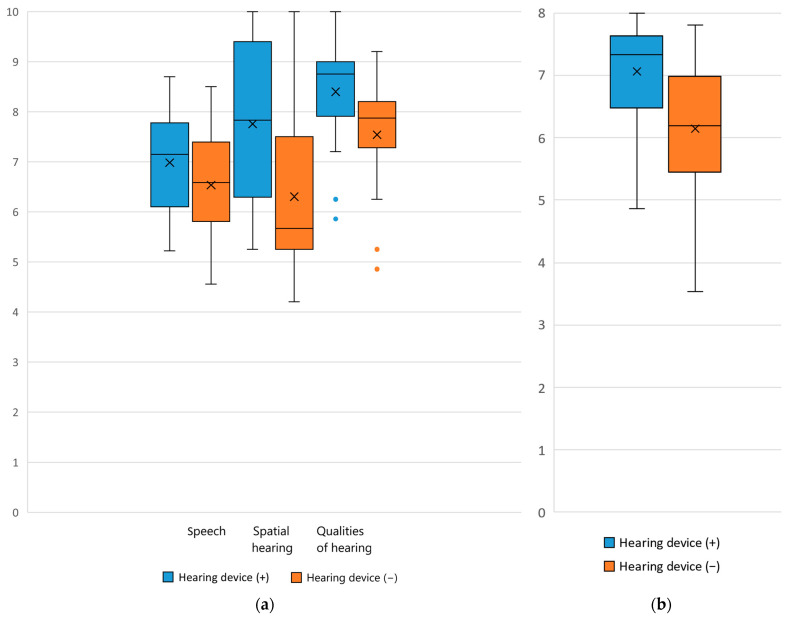
Results of SSQ and CHILD questionnaire for parents. (**a**) SSQ questionnaire; (**b**) CHILD questionnaire. The lines in the box represent the Mean, while the “x” represents the Median. The orange and blue dots are outliers.

The SSQ questionnaire showed a significant difference in daily function when listening with and without the hearing device. The median total scores were 7.8 (IQR: [6.8, 8.7]) with the device and 6.7 (IQR: [6.3, 7.6]) without the device (*p* < 0.001), while the median subscales scores were respectively 7.2 (IQR: [6.1, 7.8]) vs. 6.6 (IQR: [5.9, 7.4]) in speech scale (*p* = 0.0166), 7.8 (IQR: [6.3, 9.3]) vs. 5.7 (IQR: [5.3, 7.5]) in spatial hearing scale (*p* < 0.001), and 8.8 (IQR: [8.0, 9.0]) vs. 7.9 (IQR: [7.4, 8.2]) in qualities of hearing (*p* < 0.001). A higher score designates better hearing quality (Figure 1a). Moreover, the results were similar when comparing the abilities without device before and after the treatment (Figure 1a).

The CHILD questionnaire also highlighted significant results. The median CHILD score with the device was 7.3 (IQR: [6.5, 7.6]) and 6.2 (IQR: [5.5, 6.9]) without the device This gave a median improvement of 1 point in CHILD scores following amplification, which was statistically significant (*p* < 0.001) (Figure 1b).

Additionally, from power analysis, we observed that all significant tests had low probability of bias due to small sample size, apart from the significant test obtained from section A of the SSQ questionnaire.

## 4. Discussion

This prospective longitudinal 1 year study demonstrated the benefits for listening ability and quality of life in 18 children between 3 and 17 years with a diagnosis of unilateral sensorineural HL. They underwent UHL rehabilitation, comparing the outcomes before and after the use of a hearing device (HA or CI). The evaluation of audiological outcomes of sound field thresholds and speech perception scores, based on unaided and aided assessment (mean PTA: 79.2 ± 23.1 dB vs. 48.4 ± 14.5 dB; mean SRT at speech audiometry: 77.9 ± 29.3 dB vs. 41.3 ± 17.7 dB; mean SRT at SiIMax: −2.6 ± 2.5 vs. −3.2 ± 2.2), shows improvements in audiometric thresholds and demonstrates how the hearing devices adequately support listening and spoken language after 6 months of treatment. Scores with hearing devices were significantly higher than baseline-only scores when averaging both SSQ and CHILD questionnaires, pointing to an overall rehabilitative benefit.

The evaluation of audiological outcomes in children with UHL based on aided sound field thresholds and speech perception assessment versus unaided audiogram, before and after 6 months from hearing device fitting, shows improvements in audiometric thresholds and demonstrates how the hearing devices adequately support listening and spoken language. Interestingly, even the scores obtained by using SSQ and CHILD questionnaires were significantly improved in children who underwent hearing rehabilitation (median CHILD: 6.2 [5.5, 6.9] vs. 7.3 [6.5, 7.6]; median SSQ global score: 6.7 (IQR: [6.3, 7.6] vs. 7.8 [6.8, 8.7]; median SSQ speech scale: 6.6 [5.9, 7.4] vs. 7.2 [6.1, 7.8]; median SSQ spatial hearing scale: 5.7 [5.3, 7.5] vs. 7.8 [6.3, 9.3]; median SSQ qualities of hearing scale: 7.9 [7.4, 8.2] vs. 8.8 [8.0, 9.0].

Recent studies reveal the profound impact of UHL, with a wide range of consequences on cognitive, emotional, and social development, demanding a reassessment of clinical assumptions and management strategies. According to the literature [9,10,11,12,13,14,15,16,24,25,26,27,28], our findings support the critical need for early detection and targeted interventions.

Nowadays, despite clinical recommendations, no guidelines are accessible to improve hearing for patients with UHL and routine surveillance of speech-language, auditory and academic development are still suggested. In this context, the effectiveness of UHL management options is still challenging.

In our sample, universal newborn hearing screening detected half of prelingual children (4/8 cases), whereas audiological surveillance was crucial for the early diagnosis of late onset hearing impairment. This was the case for a baby with congenital cytomegalovirus infection diagnosed at 6 months of age and for three other cases with language delay in the absence of audiological risk factors. The universal newborn hearing screening program has become standard of care in our region since 2006 with a continuous improvement in the performance of this program over the last years [36].

However, the families were unlikely to have refused rehabilitative treatment when they received the diagnosis. In our experience, this delay after the age of 4 years or later was related to the low awareness of emerging difficulties in childhood.

The benefits of early rehabilitation are an emerging issue considering the new evidence of neural implications of UHL, as shown by neuroimaging and functional techniques [14,37]. Jung et al. reported reduced connectivity between brain networks related to language comprehension and executive functioning in UHL patients [38].

Neuroimaging research provides further evidence of these changes, revealing atypical activity and organization in auditory and prefrontal areas of children with UHL [39,40]. For instance, cortices associated with spatial hearing and binaural integration, such as the superior temporal gyrus, exhibit reduced activity, impacting on sound localization capabilities. Such deficits complicate basic auditory tasks and hinder social and academic participation. Early deprivation of auditory stimuli appears to increase the brain’s reliance on compensatory visual and somatosensory inputs, thus reinforcing maladaptive neural networks over time [41,42].

Remarkably, functional brain imaging methods, such as electroencephalography (EEG) and magnetic resonance imaging (MRI), improved the knowledge of brain connectivity, along with a new technique, near-infrared spectroscopy (NIRS). This non-invasive functional imaging technique permits detection of an increase in blood flow to active cortical areas by assessing the concentrations of oxyhemoglobin and deoxyhemoglobin when investigating cortical reorganization. In humans, the cortical reorganization that results from UHL has been investigated using different functional brain imaging methods, such as electroencephalography (EEG), magnetic resonance imaging (MRI), and more recently near-infrared spectroscopy (NIRS), which assesses cortical activity levels using spectroscopy; specifically, it detects an increase in blood flow to active cortical areas by assessing the concentrations of oxyhemoglobin and deoxyhemoglobin. In patients with UHL, a recent report suggests a correlation of this reorganization with psychoacoustic and psychosocial performance in developing rehabilitation programs [43]. Therefore, NIRS could be a frontier technique to promote tailored auditory rehabilitation programs and to prevent long-term neurofunctional and clinical consequences.

Remarkably, we observed that SSQ and CHILD questionnaires were highly effective counseling tools for parents, facilitating an understanding of listening difficulties and their potential consequences. Their routine use appears essential for fostering parental empowerment and ensuring adherence to treatment protocols. The role of family-centered approaches is an integral part of UHL management in order to better stimulate the child, confirm improvements and facilitate integration in academic environments, where children with UHL are often at a disadvantage due to teacher neglect of their specific needs [35]. Therefore, the role of the questionnaire is useful in motivating families not to postpone the intervention program in order to avoid the consequence of auditory deprivation. Bagatto et al. in their recommendations to audiologists for the management of children with UHL emphasize the importance of counseling families as an essential component of the effective care of these children [9]. Specifically, a comprehensive counseling, including a discussion of hearing technologies, implications of no treatment, expectations, and auditory training, is critical to optimizing therapeutic outcomes.

In our setting, the SSQ detected better results in spatial hearing and qualities of hearing scales, rather than the speech scale. These data agree with the better results for aided sound field audiometry compared to the unaided threshold, although the moderate hearing improvement with a mean aided PTA was 48.4 ± 14.5 dB nHL. Furthermore, this outcome seems to be mainly due to the lower amplification parameters (especially gain) in all patients, particularly in those undergoing their first prosthetic application, to avoid distortive effects. The goal of the hearing devices in these cases should not be considered as restoring the audiometric threshold, as the hearing-impaired ear should only serve to support the healthy ear in binaural listening, improving directional hearing, which was obtained in all cases, although 3/9 children with severe to profound UHL loss (i.e., SSD) opted for CI.

Moreover, it is well kwon that UHL mainly affects hearing perception in complex environments, such as speech in noise [44]. Our data show a global improvement in speech recognition in noise as confirmed at the SiIMax test, with a significant decrease for SRT between UHL children with and without a hearing device.

Both HA and CI offer potential strategies for reducing the adverse effects of UHL; however, a critical point is the reported number of non-users of devices. In our cohort, the subjective benefit of wearing a hearing device was also indirectly proven by the high mean daily device use, which was more than 8 h per day in 17/18 patients. Thus, the best results in term of speech perception and sound localization were dependent on long time usage and prolonged daily use of the device [18].

The main factors affecting the use of hearing devices in the pediatric population with UHL are discomfort, poor perception of its benefits and social stigma [45]. Additionally, a common issue that may contribute to their non-use is the lack of awareness among parents, as this solution had not been previously suggested by healthcare professionals. This factor highlights the need for developing standardized guidance from audiology services on managing pediatric unilateral sudden sensorineural hearing loss (USNHL).

In our opinion, the intervention treatment should start immediately after the diagnostic work-up to avoid the consequences of hearing deprivation in both congenital and late-onset hearing loss. However, the first line of treatment should always be rehabilitation with HA, even in SSD, and the suggestion of cochlear implantation should be deserved in SSD patients without benefit from HA, with high daily use for at least 6 months.

Accordingly, in the recent literature on children with substantial UHL or SSD, CI is recommended in improving speech perception in quiet and noise, spatial localization and for overall quality of life [46].

Specifically, early implantation in young children with SSD can rapidly restore bilateral auditory input to the cortex, needed to improve binaural hearing, allowing children to develop auditory–linguistic skills closer to those of their normal-hearing peers [47]. Thus, the overall data on hearing management and the results of SSQ and CHILD questionnaires can be useful in decision-making of CI.

In fact, in our setting, parents reported a reduction in communication difficulties, and they were more prone to accept the rehabilitative approach, confirming the role of family-centered approaches as an integral part of UHL management.

### Implications for Future Research and Clinical Practice

Despite the increasing evidence on the role of hearing rehabilitation in UHL, its management remains challenging. Our findings suggest the need for enhanced awareness among pediatricians, audiologists, and educators regarding the impacts of UHL in childhood. A standardized protocol for the identification and monitoring of UHL is fundamental, given that many children with mild or moderate HL often remain undiagnosed until developmental deficits surface. Universal newborn screening programs have demonstrated efficacy in reducing diagnostic delays [28], yet comprehensive diagnostic tools tailored to varying degrees of UHL are still lacking.

Future studies should prioritize longitudinal designs to assess the long-term outcomes of intervention strategies on auditory and psychosocial development. Additionally, emerging rehabilitative technologies, including binaural hearing systems and neurofeedback interventions aimed at rebalancing neural networks, warrant in-depth exploration. The incorporation of family training programs into intervention protocols is also essential, given their centrality in supporting children’s management of UHL at home and school [20].

## 5. Limitations

The small sample size in this study was due to the small number of children undergoing CI and HA in a single hospital. To reduce statistical biases for the obtained results, we performed a power analysis with the effect size. Particularly, the effect size confirmed all significant results obtained, minimizing the likelihood of statistical biases due to the small sample. Therefore, this study provided preliminary results that should be confirmed by a multicenter study with a large sample size, and considering a longer follow-up for each patient. The choice of a preliminary study was suggested by the need to reduce costs before carrying out a prospective study on a very large sample.

In our study, tonal and speech audiometry with masking were primarily used to optimize the fitting of hearing devices, rather than strictly assess the improvement of hearing, while the Matrix test provided insight into the “binaural” improvement achieved with the device. This kind of assessment will provide benefit derived from the device in the poorer ear, without considering binaural listening for all audiological assessments. Therefore, it could be useful to assess the two ears together, varying the source of noise and signal to obtain a more complete understanding of the binaural benefit provided by the hearing device.

## 6. Conclusions

UHL in children manifests as a complex condition with profound implications for cognitive, linguistic and social development. Although often underdiagnosed or dismissed as a mild concern, the evidence underscores its long-term impact on functional capabilities and quality of life. Rehabilitative interventions, both HA and CI, offer notable benefits when introduced early, but achieving optimal outcomes requires a multidisciplinary and individualized approach. The development of the parents’ subjective perception of the children’s quality of hearing by using questionnaires is essential to foster the counselling of parents and caregivers in promoting adherence to the appropriate intervention and rehabilitation and to monitor its outcomes, in combination with the speech in noise test. This study demonstrated statistically significant benefits of HA and CI use on hearing outcomes in children with UHL.

Further quantitative and qualitative research is desirable with a larger sample size to provide further evidence on the rehabilitative strategies of UHL.

## Figures and Tables

**Table 1:** Demographic and audiological characteristics in our sample.

Parameters	Sample
Children	18
*Age at diagnosis* (*months*)	
Mean ± SD	48.9 ± 60.2
Median (IQR)	42.0 (1.0, 72.0)
*Gender*	
Male	61.1% (11)
Female	38.9% (7)
*Age at the rehabilitation* (*months*)	
Mean ± SD	103.3 ± 49.2
Median (IQR)	96.0 (72.0, 144.0)
*Site*	
right	38.9% (7)
left	61.1% (11)
*Hearing loss degree*	
mild	5.6% (1)
moderate	16.7% (3)
severe	33.3% (6)
profound	44.4% (8)
*Device type*	
hearing aid	83.3% (15)
cochlear implant	16.7% (3)

SD = standard deviation; IQR = interquartile interval.

**Table 2:** Audiological results for the patients.

PT	AGE Diagnosis	AGE Rehab	SEX	SIDE	DEVICE	PTA (−)	PTA (+)	SRT (−)	SRT (+)	SRT SiIMAX (−)	SRT SiIMAX (+)
1	1	48	M	R	HA	82.5	68.33	NA	NA	NA	NA
2	1	96	F	L	HA	63.75	28.75	65	20	−2.4	−3.3
3	72	96	M	R	CI	120	37.5	70	25	−5.2	−3.8
4	6	84	M	L	HA	55	45	60	50	1.3	0.1
5	204	204	F	R	HA	61.25	50	60	55	NA	NA
6	1	72	F	L	HA	85	70	80	70	2	0.6
7	48	48	M	L	HA	75	51.25	NA	NA	NA	NA
8	72	72	M	R	HA	58.75	47.5	NA	NA	−0.1	−1.4
9	180	192	M	L	CI	120	37.5	NA	NA	−6.6	−7.1
10	1	36	F	L	HA	76.25	32.5	85	20	NA	NA
11	84	144	M	R	HA	98.75	78.75	NA	NA	−4.7	−3.8
12	1	144	M	L	CI	120	41.25	NA	65	−2.8	−3.9
13	60	144	F	R	HA	77.5	50	NA	45	−1.6	−3
14	1	48	F	L	HA	40	25	NA	NA	NA	NA
15	48	120	M	R	HA	61.25	55	NA	45	−2.4	−1.9
16	60	72	F	L	HA	83.75	50	NA	25	−3.4	−2.9
17	4	108	M	L	HA	81.25	62.5	25	25	−4.6	−5.7
18	36	132	M	L	HA	66.25	41.25	70	50	−3.5	−5.6

Abbreviations in the table: PT: patient; M: male; F: female; L: left; R: right; HA: hearing aid; CI: cochlear implant; PTA: pure tone average; SRT: speech reception threshold at speech audiometry; SRT SiIMAX: speech reception threshold at simplified Italian matrix; (−): without hearing device (HA/CI); (+): with hearing device (HA/CI); NA: not applicable.

**Table 3:** Results after rehabilitative treatment with and without hearing device at audiological evaluation and questionnaires.

Children After the Rehabilitation	Group 1	Group 2	Group 1 vs. Group 2	
Parameters	With Hearing Device	Without Hearing Device	*p*-Value (test)	Effect Size
PTA at pure tone audiometry			<0.0001 * (W)	*r* = 0.92 large effect
Mean ± SD	48.4 ± 14.5	79.2 ± 23.1		
Median (IQR)	48.8 [37.5, 55.0]	76.9 [61.3, 85.0]		
SRT at speech audiometry	n = 12	n = 12	0.0018 * (T)	d = 1.18 large effect
Mean ± SD	41.3 ± 17.7	77.9 ± 29.3		
Median (IQR)	46 [25.0, 52.5]	70 [60.0, 102.0]		
*SRT at SiIMax*	n = 13	n = 13	0.068 (T)	d = 0.56 medium effect
Mean ± SD	−3.2 ± 2.2	−2.6 ± 2.5		
Median (IQR)	−3.3 [−4.3, −1.8]	−2.8 [−4.6, −1.2)		
CHILD Global score			<0.0001 *(W)	*r* = 0.63 large effect
Mean ± SD	7.1 ± 0.8	6.1 ± 1.1		
Median (IQR)	7.3 [6.5, 7.6]	6.2 [5.5, 6.9]		
SSQ				
Section A (Speech)			0.0166 * (W)	*r* = 0.27 small effect
Mean ± SD	7.0 ± 1.0	6.5 ± 1.0		
Median (IQR)	7.2 [6.1, 7.8]	6.6 [5.9, 7.4]		
Section B (Spatial Hearing)			<0.0001 *(W)	*r* = 0.61 large effect
Mean ± SD	7.8 ± 1.7	6.3 ± 1.7		
Median (IQR)	7.8 [6.3, 9.3]	5.7 [5.3, 7.5]		
Section C (Qualities of Hearing)			<0.0001 *(W)	*r* = 0.61 large effect
Mean ± SD	8.4 ± 1.1	7.5 ± 1.1		
Median (IQR)	8.8 [8.0, 9.0]	7.9 [7.4, 8.2]		
Global score			<0.0001 *(W)	*r* = 0.51 large effect
Mean ± SD	7.7 ± 1.2	6.8 ± 1.1		
Median (IQR)	7.8 [6.8, 8.7]	6.7 [6.3, 7.6]		

SD = standard deviation; IQR = interquartile interval; * = significant test; T = paired *t*-test; W = Wilcoxon test.

## Data Availability

The original contributions presented in the study are included in the article, further inquiries can be directed to the corresponding author.

## Abbreviations

The following abbreviations are used in this manuscript:

UHL	Unilateral hearing loss
HL	Hearing loss
HA	Hearing aid
CI	Cochlear implant
WHO	World Health Organization
PTA	Pure tone average
SSD	Single-sided deafness
CHILD	Children’s Home Inventory for Listening Difficulties
SSQ	Speech and Qualities of Hering Questionnaire
SiIMax	Simplified Italian matrix
SRT	Speech recognition threshold
SD	Standard deviation
IQR	Interquartile range
NIRS	Near-infrared spectroscopy
